# Case Report: Camrelizumab plus gemcitabine followed by concurrent chemoradiation for HPV-positive and PD-L1-positive tonsillar squamous cell carcinoma

**DOI:** 10.3389/fimmu.2025.1528198

**Published:** 2025-03-05

**Authors:** Xuesong Li, Bianhong Wang, Hao Liu, Zhuo Yu

**Affiliations:** ^1^ Department of Medical Oncology, Beijing Tsinghua Changgung Hospital, School of Clinical Medicine, Tsinghua University, Beijing, China; ^2^ Department of Hematology, Beijing Tsinghua Changgung Hospital, School of Clinical Medicine, Tsinghua University, Beijing, China; ^3^ Department of Pathology, Beijing Tsinghua Changgung Hospital, School of Clinical Medicine, Tsinghua University, Beijing, China

**Keywords:** tonsillar squamous cell carcinoma, camrelizumab, gemcitabine, chemoradiation, treatment

## Abstract

Modality treatment including surgery, chemotherapy, radiation and immunotherapy was considered as standard strategy for tonsillar squamous cell carcinoma (TSCC). We reported a case of a 78-year-old man under severe underlying medical conditions who diagnosed with human papillomavirus (HPV)-positive and programmed death ligand 1 (PD-L1)-positive TSCC. He was administered camrelizumab combined with gemcitabine (GEM) for 8 cycles with partial response. Then, we gave him concurrent nanoparticle albumin-bound paclitaxel (nab-paclitaxel)-based chemoradiation. Eventually, this patient achieved complete response without severe adverse events. After a 12-month follow-up, he had no recurrence or metastasis.

## 1 Introduction

Tonsillar squamous cell carcinoma (TSCC) constituted the predominant portion of oropharyngeal cancers, representing 15%-20% of oropharyngeal squamous cell carcinoma (OPSCC) cases, and exhibited a high prevalence of human papillomavirus (HPV) positivity (1). The majority of patients with TSCC who underwent multimodal therapy experienced favorable outcomes compared to those who received a single treatment scheme (2). HPV-driven head and neck squamous cell carcinoma (HNSCC) harbored a distinct immune microenvironment including increased programmed death ligand 1 (PD-L1) expression and activated CD8 + T cells (3). A recent study found that patients with HPV-positive HNSCC treated with the inhibitor of programmed cell death protein 1 (PD-1)/PD-L1 showed greater objective response rate than those with HPV-negative HNSCC (4). Camrelizumab, a PD-1 inhibitor, has demonstrated promising efficacy and acceptable safety in HNSCC patients (5). Gemcitabine (GEM) exerted anti-tumor function by inhibiting DNA synthesis, with HNSCC being one of its target cancers. Moreover, several studies have found that GEM can induce PD-L1 expression and exhibit immunomodulatory effects without compromising cellular immunity (6, 7). In the manuscript, we reported a 78-year-old man with HPV-positive and PD-L1-positive TSCC who received camrelizumab plus GEM followed by concurrent chemoradiation and achieved complete response (CR).

## 2 Case report

**Figure 1:**
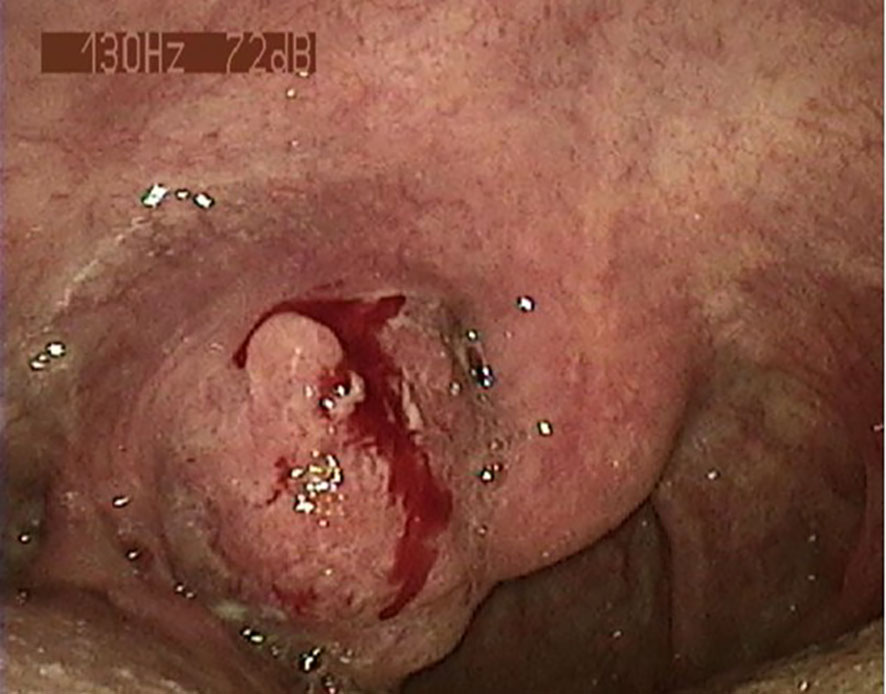
The right tonsil prone to bleed was observed by fibrolaryngoscopic examination.

**Figure 2:**
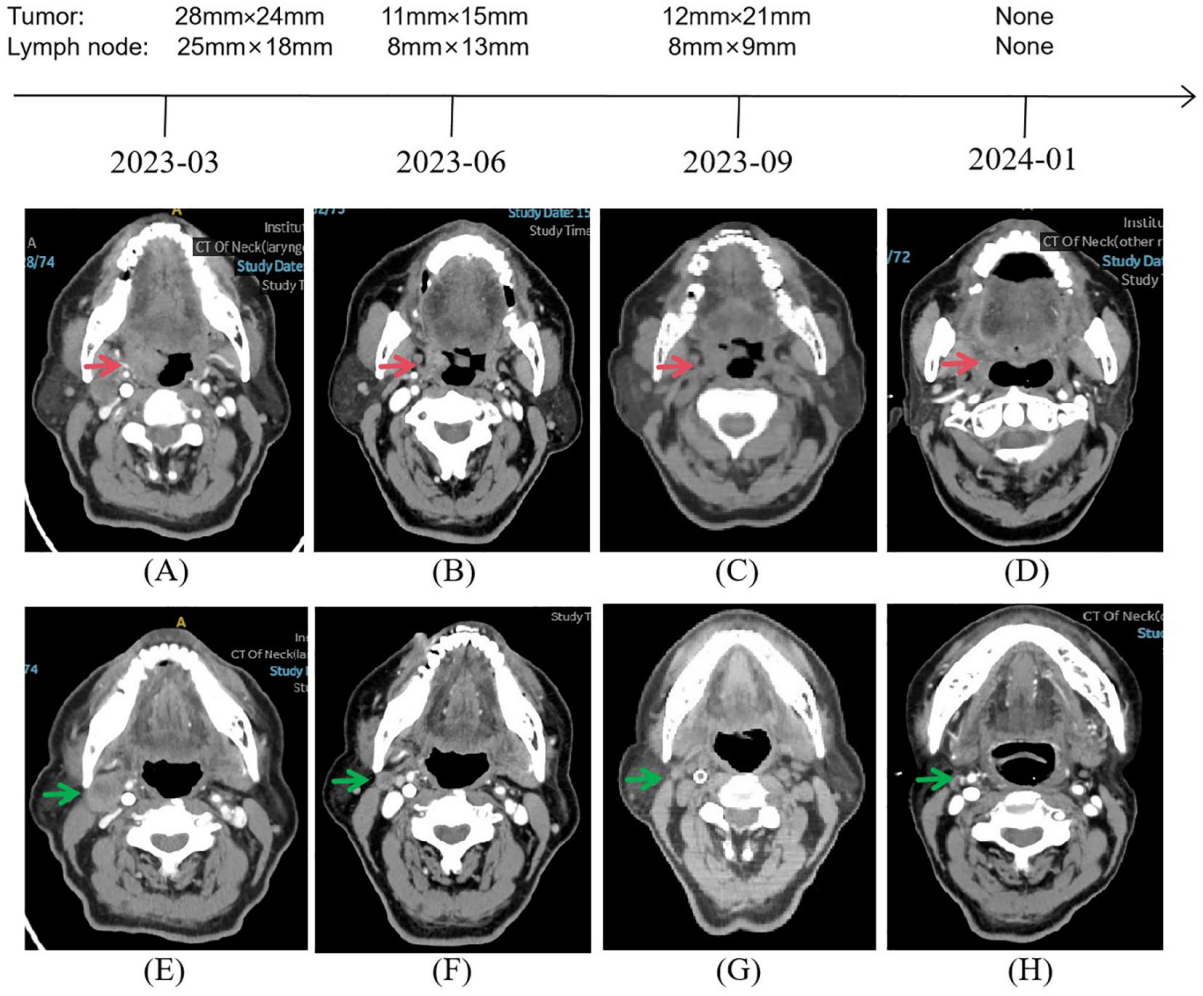
Representative images during the whole treatment. CT imaging exhibited a continued partial response after a total of 8 cycles of camrelizumab plus gemcitabine from March 2023 to September 2023, followed by a complete response under concurrent chemoradiotherapy based on nab-paclitaxel from September 2023 to January 2024. Red arrow showing the tumor. Green arrow showing the neck lymph node.

**Figure 3:**
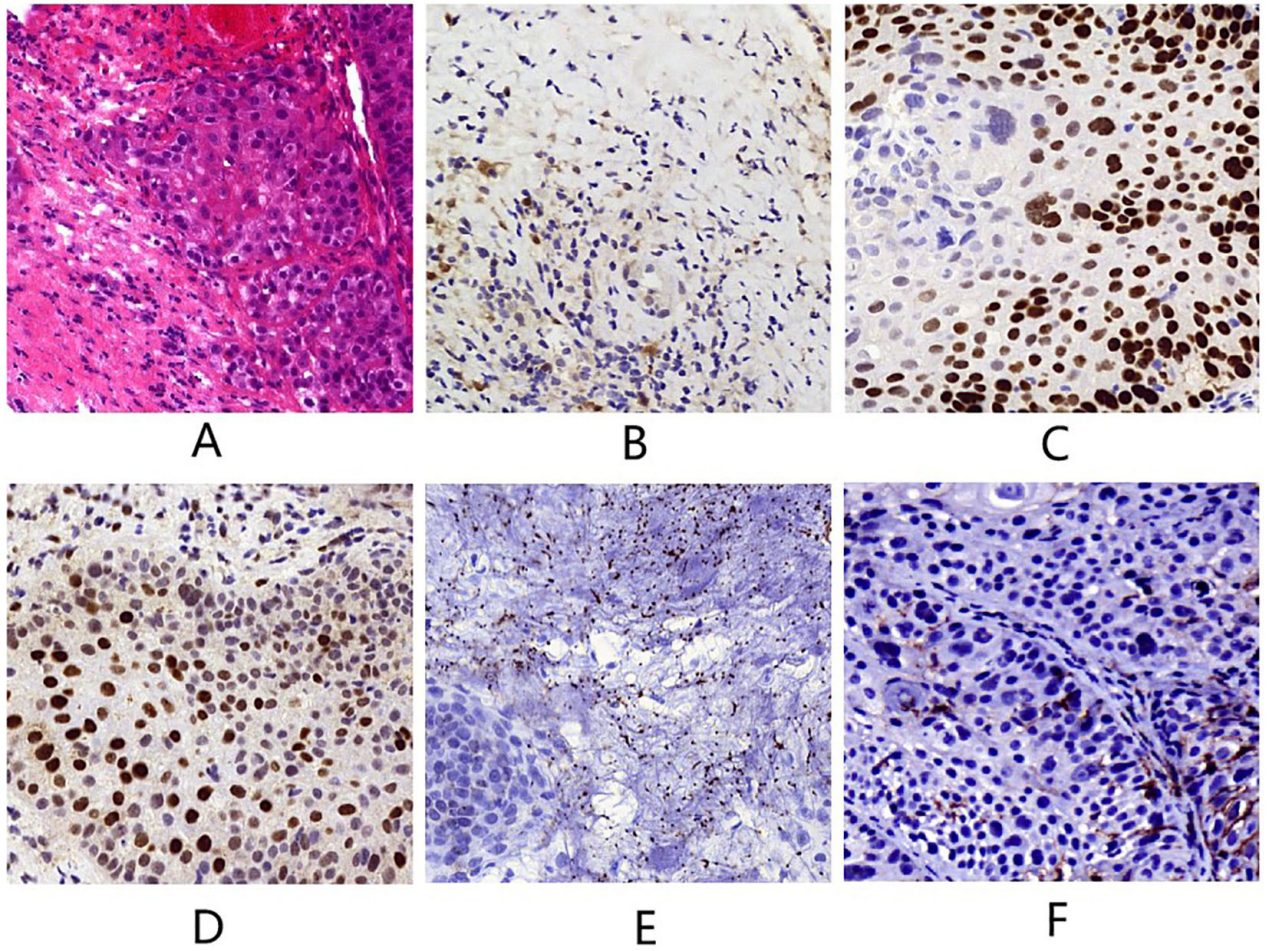
**(A)** Tumor tissue (hematoxylin and eosin staining, magnification×400). **(B)** p16 (magnification×400). **(C)** p40 (magnification×400). **(D)** p53 (magnification×400). **(E)** HPV-RNA (magnification×400). **(F)** PD-L1 (magnification×400).

A 78-year-old man presented to our outpatient clinic with complaint of oropharyngeal discomfort for 4 months. Intraoral examination indicated a swollen tonsil covered with exudate. No abnormalities were observed in the left tonsil. Locoregional physical examination revealed a large mass of the right submandibular region. There is no any deficits in the function of cranial nerve. He had a history of coronary artery disease, right carotid artery stenosis, right vertebral artery occlusion, acute kidney injury, hypertension, and hyperlipemia. He denied having a family history of cancer. Fibrolaryngoscopic examination showed that the right tonsil was prone to bleeding and had an uneven surface (**Figure 1**). The computed tomography (CT) scan of neck revealed a round soft-tissue density mass of right tonsil (28mm) and a metastastic neck lymph node (18mm) in March 2023 (**Figures 2A, E**). TSCC was confirmed by histopathological analysis of a biopsy sample from the mass with the positivity for p16, p40, and p53 (**Figures 3A–D**). The ki67 proliferation index was approximately 80%. HPV-RNA of tissue was detected by in situhybridization method (**Figure 3E**). PD-L1 expression was markedly positive with a combined positive score of 10 and a tumor proportion score of 3% (**Figure 3F**). The multi-disciplinary team discussed the conclusion that the surgery risk of the patient was extremely high due to severe underlying medical conditions and the PD-1 inhibitor combined with GEM may be an optimal choice. On April 4, 2023, this patient was administered intravenously camrelizumab (200 mg per 3 weeks) and GEM (1.6g once, days 1 and 8, 21 days as a cycle). After 4 cycles of treatment, both the tumor and lymph node sizes were dramatically reduced (tumor: from 28 to 15 mm, lymph node: from 18 to 8 mm) with partial response (PR) according to RECIST criteria (**Figures 2B, F**) in June 2023. After a total of 8 cycles of treatment, the CT scan revealed that the size of tumor was slightly increased (from 15 to 21 mm) whereas the size of lymph node was decreased in September 2023 (**Figures 2C, G**). This patient still experienced PR. Considering the enlarged tumor, we gave him radiotherapy with a dose of 54Gy/30f and concurrent nanoparticle albumin-bound paclitaxel (nab-paclitaxel) intravenously (100 mg per week, total 4 weeks). On January 3, 2024, CT revealed that both the tumor and the lymph node were diminished and this patient achieved CR (**Figures 2D, H**). After a 12-month follow-up, he had no recurrence or metastasis.

## 3 Discussion

Anti-PD-1 strategies have been extensively developed in tumor immunotherapy, particularly in solid tumors (8). PD-1 inhibitor has been proved to be an effective first-line therapy for PD-L1-expressing HNSCC patients in recent KEYNOTE-048 trial (9). In a phase II study, nivolumab combined with GEM showed encouraging outcomes for patient with recurrent or metastatic HNSCC (10). In this case, this patient managed to achieve PR although the tumor had a tendency of enlargement after receiving camrelizumab plus GEM. Then, he was administered concurrent chemoradiotherapy, and had CR without severe adverse events. This patient’s liver and kidney functions were normal after treatment. Studies revealed that GEM induced squamous cell carcinoma radiation enhancement through modulating of cell cycle relying on exposure duration, drug concentration, and combined treatment regime (11). Nab-paclitaxel is a suspension comprising albumin-bound nanoparticle of paclitaxel, which exhibited superior clinical efficacy and safety compared to paclitaxel despite containing the same active ingredient. It has been demonstrated that nab-paclitaxel displayed inspiring response rates on patients with locally advanced oropharyngeal cancer (12). However, whether camrelizumab plus GEM enhanced the antitumor effect of chemoradiotherapy based on nab-paclitaxel remained elusive. The mechanisms underlying camrelizumab plus GEM followed by concurrent chemoradiotherapy treatment for HPV-positive and PD-L1-positive TSCC warrant further exploration.

Most HPV-positive OPSCC patients are young males, while HPV-negative OPSCC patients are typically older (13). However, the incidence of OPSCC is rising among elderly patients in the United States, primarily attributed to the growing prevalence of HPV-associated cancers (14). A retrospective study of 113 elderly patients with HPV-positive OPSCC reported that 3-year and 5-year overall survival (OS) were 62.4% and 50.4%, respectively. Furthermore, elderly patients exhibited distinct clinicopathological characteristics, and factors such as T stage and chemotherapy can served as independent predictors for OS (15). A study including HPV-positive oropharyngeal cancer patients aged 65 and older demonstrated that elderly individuals showed favorable OS and high treatment tolerance, with a 3-year OS rate of 85.5% and a 3-year disease-free survival rate of 67.3% (16). Given the distinct challenges in treating elderly patients with OPSCC, further studies should investigate the applicability of the therapeutic strategy in diverse populations. Although the 12-month follow-up provides preliminary data, we recognize that it falls within a high-risk period for recurrence. The need for follow-up to better understand the long-term outcomes is warrant.

The NCCN guidelines suggested that patients unable to tolerate surgery should prioritize participation in clinical trials, and the CSCO guidelines recommended radiation therapy alone for patients with locally advanced disease who do not meet criteria for surgery (17, 18). GEM can serve as a PD-L1 upregulation agent, enhancing the efficacy of camrelizumab by increasing PD-L1 expression, which in turn amplified the immune response. Additionally, GEM promoted T-cell killing by increasing MHC-I expression on tumor cells and selectively reducing the number of myeloid-derived suppressor cells. This synergistic effect between camrelizumab and GEM can lead to a more robust antitumor immune response (19). Furthermore, a phase II clinical trial revealed that the combination of camrelizumab with GEM and oxaliplatin was well-tolerated in patients with classical Hodgkin lymphoma (20). Research has demonstrated that GEM has radiopotentiation effects, with its impact varying in a dose-dependent and time-dependent manner across different cell lines (21). Mechanistically, GEM can induce cell accumulation at more radiosensitive cell cycle phases, such as the G1/S transition, which facilitated the cytotoxicity of radiation therapy. However, the duration for which GEM maintains its radiopotentiation effects following chemotherapy is not well established. Camrelizumab plus GEM combined with chemoradiotherapy in HPV-positive TSCC is noteworthy due to its potential to enhance antitumor effects, improve safety and tolerability for patients with comorbidities, and contribute to the development of more personalized treatment strategies.

Between June and September 2023, the patient continued the same treatment regimen that had initially shown a partial response. Several factors may have contributed to the tumor size increase. Firstly, the tumor may have developed resistance to the administered therapies, as cancer cells can adapt and evolve to evade the effects of chemotherapy and immunotherapy (22). Additionally, the patient’s general health condition and immune status likely played a role in shaping the treatment response. Variations in immune function have the potential to undermine the effects of immunotherapy, thereby facilitating tumor progression (23). We previous reported a woman with von Hippel-Lindau disease who initially received sorafenib and achieved PR (8). However, her tumor showed a tendency to grow, but this enlargement was successfully managed through the combination of sorafenib and a PD-1 inhibitor. Wang et al. reported a patient who exhibited initial promising results with PD-1 inhibitor combined with chemotherapy. After 129 days of treatment, disease progression occurred due to resistance (24). In brief, HPV-positive and PD-L1-positive TSCC represented a distinct subtype of HNSCC, and camrelizumab plus GEM followed by concurrent chemoradiotherapy could be a promising therapeutic strategy for HPV-positive and PD-L1-positive TSCC.

## Data Availability

The original contributions presented in the study are included in the article/supplementary material. Further inquiries can be directed to the corresponding author/s.
